# Is a Severe Clinical Profile an Effect Modifier in a Web-Based Depression Treatment for Adults With Type 1 or Type 2 Diabetes? Secondary Analyses From a Randomized Controlled Trial

**DOI:** 10.2196/jmir.1657

**Published:** 2012-01-05

**Authors:** Kim MP van Bastelaar, François Pouwer, Pim Cuijpers, Heleen Riper, Jos WR Twisk, Frank J Snoek

**Affiliations:** ^1^Department of Medical PsychologyVU University Medical CentreAmsterdamNetherlands; ^2^EMGO+ Institute for Health and Care ResearchVU University Medical CentreAmsterdamNetherlands; ^3^Centre of Research on Psychology in Somatic Diseases (CoRPS)Department of Medical Psychology & NeuropsychologyUniversity of TilburgTilburgNetherlands; ^4^Department of Clinical PsychologyVU UniversityAmsterdamNetherlands; ^5^EMGO+ Institute for Health and Care ResearchVU UniversityAmsterdamNetherlands; ^6^GGZinGeestAmsterdamNetherlands; ^7^Institute of PsychologyLeuphana UniversityLüneburgGermany; ^8^Department of Epidemiology and BiostatisticsVU University Medical CentreAmsterdamNetherlands; ^9^Faculty of Health SciencesVU UniversityAmsterdamNetherlands

**Keywords:** Diabetes mellitus, type 1, diabetes mellitus, type 2, depression, behavior therapy, cognitive therapy, depressive disorder, adults, psychology

## Abstract

**Background:**

Depression and diabetes are two highly prevalent and co-occurring health problems. Web-based, diabetes-specific cognitive behavioral therapy (CBT) depression treatment is effective in diabetes patients, and has the potential to be cost effective and to have large reach. A remaining question is whether the effectiveness differs between patients with seriously impaired mental health and patients with less severe mental health problems.

**Objective:**

To test whether the effectiveness of an eight-lesson Web-based, diabetes-specific CBT for depression, with minimal therapist support, differs in patients with or without diagnosed major depressive disorder (MDD), diagnosed anxiety disorder, or elevated diabetes-specific emotional distress (DM-distress).

**Methods:**

We used data of 255 patients with diabetes with elevated depression scores, who were recruited via an open access website for participation in a randomized controlled trial, conducted in 2008–2009, comparing a diabetes-specific, Web-based, therapist-supported CBT with a 12-week waiting-list control group. We performed secondary analyses on these data to study whether MDD or anxiety disorder (measured using a telephone-administered diagnostic interview) and elevated DM-distress (online self-reported) are effect modifiers in the treatment of depressive symptoms (online self-reported) with Web-based diabetes-specific CBT.

**Results:**

MDD, anxiety disorder, and elevated DM-distress were not significant effect modifiers in the treatment of self-assessed depressive symptoms with Web-based diabetes-specific CBT.

**Conclusions:**

This Web-based diabetes-specific CBT depression treatment is suitable for use in patients with severe mental health problems and those with a less severe clinical profile.

**ClinicalTrial:**

International Standard Randomized Controlled Trial Number (ISRCTN): 24874457; http://www.controlled-trials.com/ISRCTN24874457 (Archived by WebCite at http://www.webcitation.org/63hwdviYr)

## Introduction

With an estimated world prevalence of 285 million people, diabetes mellitus has reached epidemic levels globally [1]. Affecting 10% to 20% of the adult diabetes patients, depression is to be regarded as a common comorbid health problem that negatively affects quality of life and diabetes outcomes, and increases mortality [2,3]. Treating depression in diabetes is therefore of great importance.

A recent meta-analysis has shown that depression in diabetes patients can be effectively treated with various antidepressant treatments, showing the highest effect sizes for psychological treatment (Cohen *d* = –0.58, 95% CI, –0.77 to –0.39), compared with pharmacologic treatment (Cohen *d* = –0.47, 95% CI, –0.66 to –0.27) or collaborative care (Cohen *d* = –0.29, 95% CI, –0.43 to –0.16) [4].

Nevertheless, in a substantial portion of diabetes patients, comorbid depression remains untreated. Underrepresentation of complaints, underrecognition of depressive symptoms by health care providers, and inadequate referral can account for this [5,6]. Another reason for untreated depression in diabetes patients is the negative stigma of mental health care among patients who are treated in physical health care, or that they do not feel at home in a mental health care setting [7,8]. It has also been suggested that, since generic and disease-specific emotional distress are not the same, we need to tailor interventions to the specific needs of this subgroup of patients [9,10]. A depression treatment that specifically addresses elevated diabetes-specific emotional distress (DM-distress) could help overcome this last barrier to treatment. The VU University Medical Center in Amsterdam, The Netherlands, has recently developed such a cognitive behavioral therapy (CBT) depression intervention, specifically tailored to the needs of diabetes patients by incorporating diabetes-specific topics, such as coping strategies for diabetes-specific issues [11].

 Providing psychological interventions via the Internet could help overcome barriers to treatment related to travelling distance and time—for example, it has the potential to avoid reluctance to seek therapy among patients who are ashamed of needing psychological help, and it allows patients to work at home, in their own pace and familiar environment, while saving them time, and the burden and cost of travelling. Therefore, an Internet-administered intervention has the potential to have a broad reach. Internet-administered therapy can also save therapists time, thus reducing waiting lists [12]. Providing psychological interventions via the Internet can be a major advantage specifically for diabetes patients, since they already spend much time in (somatic) health care, and severe diabetes complications can cause physical impairments, causing difficulties in patients’ mobility. Considering these advantages, the diabetes-specific depression intervention, called Diabetergestemd.nl (DbG.nl), was offered via the Internet and tested in a randomized controlled trial (RCT) [13]. The DbG.nl intervention was found to be significantly more effective than a waiting-list control condition [14].

 A commonly heard criticism in studies regarding Web-based CBT depression treatment is that most studies do not differentiate between elevated symptoms of depression (subclinical depression) and diagnosed depression in the strict sense, or major depressive disorder (MDD) [15]. This causes clinicians to need more convincing evidence that online CBT can help their patients with subclinical depression, but also those with MDD, especially since the current guidelines for depression treatment indicate that patients with subclinical depression warrant a different treatment (low-intensity psychosocial interventions, such as guided self-help) from patients with MDD (high-intensity psychosocial intervention, such as individual CBT) [16]. In the RCT studying the effectiveness of DbG.nl, a diagnostic instrument was administered, which enabled us to make a clear distinction between patients with subclinical depression and patients with MDD. Examination of potential differences in effectiveness between both subgroups provides important information regarding the potential utility of the intervention from a public health perspective.

The elevated prevalence of anxiety disorders in type 1 and type 2 diabetes in comparison with prevalence rates in the general population has been demonstrated in a systematic review [17]. Since studies on the prevalence of co-occurring anxiety and mood disorders in diabetes patients have yielded mixed results [18], we were interested in exploring the co-occurrence of anxiety disorders in patients with MDD. Furthermore, the treatment literature indicates that the combination of MDD and anxiety disorder is more difficult to treat than MDD alone [19]. Therefore, we aimed to examine the effect modification of anxiety disorders in our study sample.

An important issue to consider in the context of psychological interventions for people with diabetes is the role of DM-distress and the need to accurately differentiate DM-distress from general emotional distress [20]. Previous reports have emphasized that diabetes patients with elevated symptoms of depression are not all necessarily clinically depressed, but rather may have high levels of diabetes-related distress [21,22]. Our RCT allowed us to test whether the online diabetes-specific depression intervention was more or less effective in patients with baseline elevated DM-distress than in those with lower levels of DM-distress.

To summarize, we set out to answer the following questions: does the effectiveness of a Web-based, diabetes-specific CBT depression intervention differ (1) for patients with or without MDD, (2) for patients with or without an anxiety disorder, and (3) for patients with or without elevated DM-distress? We hypothesized that Web-based, diabetes-specific CBT is more effective in diabetes patients with MDD, anxiety disorder, or elevated DM-distress.

To the best of our knowledge, this is the first study that used data from an RCT to perform secondary analyses to test effect modification in subgroups of diabetes patients regarding the effectiveness of a Web-based, diabetes-specific CBT depression treatment.

## Methods

### Participants and Procedure

**Figure 1:**
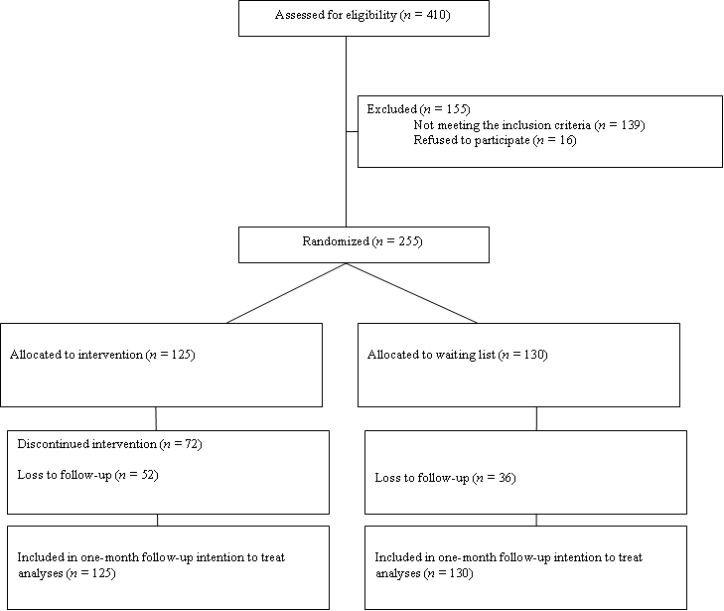
CONSORT study flowchart.

Patients for an RCT were recruited from July 2008 trough September 2009. We randomly assigned 255 adult diabetes patients with elevated depressive symptoms (having a score of 16 or higher on the Center for Epidemiologic Studies Depression scale [CES-D]) to the Web-based diabetes-specific depression intervention (n = 125) or a 12-week waiting-list control group (n = 130) (see Figure 1) [13]. Exclusion criteria were a history of suicide attempt(s) or current suicidal ideation; bipolar depression or psychotic disorder; pregnancy; recent loss of a significant other (<6 months ago); and insufficient Internet literacy. Patients were recruited via advertisements in various media and could sign up for participation in the study via an open access study website. The study was advertised as a study performed by the VU University Medical Center for testing the effectiveness of diabetes-specific online depression treatment. It was mentioned that attending was cost-free and no financial reward was provided.

 Patients could individually sign up for participation in the study through an open access study website. Written informed consent was obtained by mail and included information on the study, permission for anonymous data use, and permission to contact the patient’s general practitioner and treating diabetes physician for obtaining data on diabetes. After having signed the informed consent, patients were invited to fill out the baseline assessment through a personal online questionnaire, and they received a telephone-administered diagnostic interview.

Individual randomization by computer was used to assign participants to either the experimental or control condition, at an individual level, using a 1:1 allocation ratio. Due to the nature of the study (waiting-list controlled) it was not possible to blind patients to treatment allocation. The sample size was calculated based on the expected difference in the primary outcome variable (ie, depressive symptoms). Based on a statistical power of 80%, with an alpha of .05, we required 100 participants in each group to be able to detect differences with an effect size of 0.35. The design of the RCT on the effectiveness of the Web-based diabetes-specific depression intervention has been described in more detail elsewhere [13]. Data of the RCT were used to perform secondary analyses on effect modification. All randomly assigned participants were analyzed. The Medical Ethics Committee of the VU University Medical Center approved the study. The results of the RCT have been described elsewhere [14]. In short, the intervention was effective in reducing depressive symptoms and diabetes-specific emotional distress.

### Intervention

**Figure 2:**
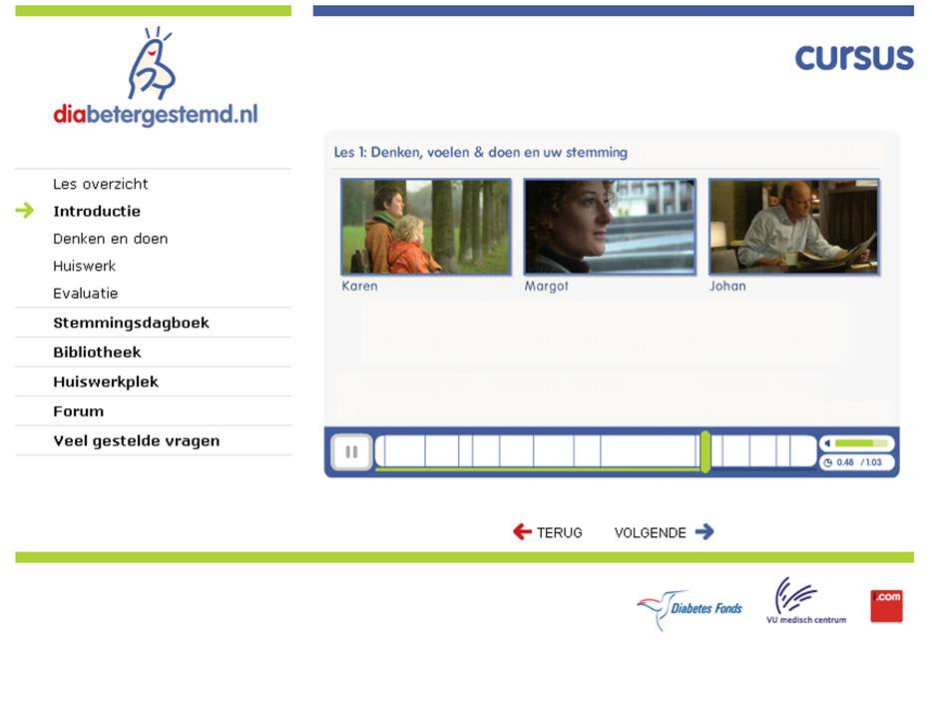
Screenshot of a lesson in the web-based diabetes-specific course.

Participants assigned to the intervention group individually attended the online course, based on the principles of CBT. The intervention DbG.nl (www.diabetergestemd.nl) was developed by the VU University Medical Center in collaboration with the Trimbos Institute. DbG.nl was based on the effective Web-based CBT depression intervention Color Your Life [22], the Internet version of Lewinsohn’s effective and well-known Coping with Depression course [23]. DbG.nl follows the same format as Color Your Life, putting emphasis on the following skills: relaxation, cognitive restructuring (including worrying), positive reinforcement, assertiveness, communication skills, and increasing the number of pleasant activities. In short, the course consists of eight consecutive weekly lessons, consisting of psychoeducation and focused on skills such as relaxation, cognitive restructuring (including worrying), positive reinforcement, social skills, and increasing the number of activities that are pleasant to the patient. The course contained written and spoken information and homework assignments (see Figure 2 for a screenshot) with one-time incorporated email feedback for each lesson from a coach (qualified psychologist). Patients were advised to go through one lesson per week. In case we did not receive their homework, patients were sent reminders after 1 week and after 2 weeks. If we received no reply within 3 weeks, we sent participants an email stating that we had to assume that they were no longer interested in the intervention, and we invited them to fill out the postmeasurement. However, if they were still interested, they were invited to reenter the course. After the course, patients were invited to fill out feedback forms, and we interviewed several patients by telephone.

Patients attended nonanonymously, so having multiple identities was not possible. Coaches only knew patients’ names, and all personal data were omitted after the patients’ medical data were obtained from their treating physician (to which the patients consented) and the participant completed or withdrew from the course. Coaches were blinded to treatment allocation of participants.

### Sample Characteristics

Baseline characteristics of our study sample were self-reported as part of the online assessment. Previous analyses confirmed successful randomization: intervention and control groups did not statistically significantly differ regarding demographic or clinical characteristics at baseline (all *P* ≥ .05) [14]. Of those randomly assigned to the intervention group, 53 (42%) completed the entire eight-lesson course, 30 (24%) completed no lesson at all, and 7 (6%) never logged into the course. Other participants dropped out equally divided during the course.

### Outcome Measure

Depressive symptoms were self-assessed online with the Dutch version of the CES-D. The CES-D is a validated self-report screening instrument that measures the frequency with which participants have experienced specific depressive symptoms within the preceding week. The questionnaire contains 20 items assessed on a 4-point Likert scale. The total score can range from 0 to 60, where higher scores indicate more depressive symptoms. In Dutch samples, a cut-off point of 16 or higher is generally accepted to indicate clinical depression [24].

### Potential Effect Modifiers

Based on our research questions, we selected the following three potential effect modifiers: MDD (yes/no), anxiety disorder (yes/no), and high versus low level of DM-distress.

To diagnose MDD and anxiety disorder, the Dutch, we administered by telephone the computerized version of the World Health Organization Composite International Diagnostic Interview (WHO CIDI-auto), a fully structured psychiatric diagnostic interview that assesses diagnostic criteria of mental disorders according to the *Diagnostic and Statistical Manual of Mental Disorders*, 4th edition. The WHO CIDI-auto is a computerized version of the WHO CIDI, a qualified substitution of the face-to-face interview [25]. Since questions and routes are fully specified, no clinical judgment is required. Interviewers were masters students in clinical psychology of the VU University in Amsterdam, trained in the administration of the WHO CIDI-auto by telephone.

 DM-distress was measured using the Dutch-validated Problem Areas in Diabetes scale (PAID), which was self-assessed online [26]. We compared the effectiveness of our intervention in patients with elevated DM-distress (using the cut-off of PAID ≥40) with those without elevated DM-distress (PAID <40).

### Statistical Analyses

To test effect modification, the course of depressive symptoms at baseline, posttreatment, and 1-month follow-up between the intervention and control groups was compared for each potential effect modifier. We performed generalized estimating equation (GEE) analyses using 3-way interaction terms (group × time × potential effect modifier) to examine whether having an MDD (yes/no) diagnosis, anxiety disorder (yes/no) diagnosis, and elevated DM-distress (PAID ≥40/<40) were significant effect modifiers in the treatment effect. All analyses were corrected for baseline depression scores to gain insight into the relative degree of change, and for time between baseline measurement and postmeasurement. Also, all potential effect modifiers were examined on baseline differences for all of the measured sociodemographic and clinical variables, and all analyses were corrected for these differences.

 In the RCT data, overall study attrition was 32% at postassessment and 35% at 1-month follow-up. Since study attrition was higher in noncompleters of the course at the 1-month follow-up than in completers of the course (63% vs 13%; *P* < .001), attrition was not completely at random. We therefore imputed missing data using the state-of-the-art multiple imputation technique with Stata 10.0 software (StataCorp LP, College Station, TX, USA). Multiple imputation minimally alters variance of data and thus provides best estimates of true data [27]. All further statistical analyses were performed using complete data, with either Stata 10.0 or SPSS 15.0 software (IBM Corporation, Somers, NY, USA). All results were based on intention-to-treat analyses.

## Results

### Baseline Characteristics

Baseline sociodemographic and clinical characteristics of our study sample are presented in Table 1, and have been described in more detail elsewhere [14].

**Table 1:** Baseline sociodemographic and clinical characteristics of study sample at baseline

Characteristics			All patients (n = 255)	CBT^a^ participants (n = 125)	Waiting-list control participants (n = 130)	*P* value
**Sociodemographics**						
	Age (years), mean (SD)		50 (12)	48 (12)	51 (12)	.51
	Women, n (%)		155 (60.7)	82 (66)	73 (56)	.12
	White, n (%)		227 (89.0)	110 (88)	117 (90)	.87
	Marital state: with partner, n (%)		199 (78.0)	99 (79)	100 (77)	.66
	Education level, n (%)					
		No formal qualifications	8 (3)	5 (5)	3 (3)	.44
		High school or lower/middle vocational qualifications	136 (53.3)	70 (56)	66 (51)	.40
		College qualifications or more	111 (43.6)	50 (40)	61 (47)	.27
**Clinical characteristics**						
	Depressive symptoms (CES-D^b^, range 16–60), mean (SD)		28 (7)	29 (7)	28 (7)	.50
	Diabetes-specific emotional distress (PAID^c^, range 0–100), mean (SD)		40 (19)	42 (19)	38 (19)	.05
	Type 2 diabetes, n (%)		141 (55)	66 (53)	75 (58)	.43
	Mean HbA_1c_ level^d^, %		7.4 (1.3)	7.4 (1.6)	7.3 (1.6)	.36
	Self-reported diabetes complications, n (%)					
		Neuropathy	25 (10)	11 (9)	14 (11)	.23
		Nephropathy	11 (4)	5 (4)	6 (5)	.69
		Retinopathy	30 (12)	17 (14)	13 (10)	.69
		Foot ulcer	21 (8)	9 (7)	12 (9)	.24
**Diagnosis of depressive disorder (WHO CIDI-auto)** **^e^** **, n (%)**						
	MDD^f^		146 (57)	71 (57)	75 (58)	.89
		MDD, single episode, mild	62 (24)	29 (23)	33 (25)	.68
		MDD, single episode, moderate	48 (19)	26 (21)	22 (17)	.43
		MDD, single episode, severe	21 (8)	9 (7)	12 (9)	.56
		MDD, recurrent episode, mild	8 (3)	4 (3)	4 (3)	1.00
		MDD, recurrent episode, moderate	4 (2)	1 (1)	3 (2)	.33
		MDD, recurrent episode, severe	3 (1)	2 (2)	1 (1)	.54
	Dysthymic disorder		28 (11)	13 (10)	15 (12)	.68
**Diagnosis of anxiety disorder, n (%)**			95 (37)	43 (34)	52 (40)	.36
	Generalized anxiety disorder		59 (23)	25 (20)	34 (26)	.24
	Social phobia		24 (9)	12 (9)	12 (9)	.92
	Panic disorder		12 (5)	4 (3)	8 (6)	.27
	Panic disorder with agoraphobia		5 (2)	3 (2)	2 (2)	.62
	Agoraphobia		11 (4)	3 (2)	8 (6)	.14
	Specific phobia		42 (16)	20 (16)	22 (17)	.83
		Blood-injection-injury type	22 (9)	15 (12)	7 (5)	.06
		Environment type	15 (6)	6 (5)	9 (7)	.47
		Situational type	9 (4)	5 (4)	4 (3)	.69
		Animal type	6 (3)	1 (1)	5 (4)	.12

^a^ Cognitive behavioral therapy.

^b^ Center for Epidemiologic Studies Depression scale.

^c^ Problem Areas In Diabetes scale.

^d^ Glycosylated hemoglobin.

^e^ World Health Organization Composite International Diagnostic Interview.

^f^ Major depressive disorder.

### Potential Effect Modifiers

As shown in Table 1, over half of the patients in our study sample (n = 146, 57.3%) had an MDD diagnosis, of whom the majority (131/146, 89.7%, had a single episode of MDD, not a recurrent depression. About half of the patients with an MDD comorbidly had an anxiety disorder (69/146, 47%) and about half had comorbid elevated DM-distress (80/146, 55%) (Table 2). Furthermore, of patients with an MDD diagnosis, a higher percentage had type 2 diabetes (94/164, 64% vs 47/109, 43%, *P* < .001) and a lower percentage used antidepressant medication (7/109, 6% vs 21/146, 14%, *P* < .001). MDD patients reported higher baseline symptoms of depression, with a mean (SD) CES-D of 30 (7) versus 26 (7), *P* < .001, and higher levels of DM-distress, mean (SD) PAID 42 (20) versus 37 (17), *P* = .02. About a third of the total study sample (n = 95, 37%) had an anxiety disorder diagnosed. Patients with an anxiety disorder diagnosis had higher baseline depressive symptoms, with a mean (SD) CES-D of 31 (8) versus 27 (7), *P* < .001, and DM-distress, mean (SD) PAID 48 (18) versus 36 (18), *P* < .001, and did not differ significantly on any sociodemographic variable.

**Table 2:** Prevalence (%) of diagnosed depression, diagnosed anxiety disorder by elevated and low diabetes-specific emotional distress ( 40 < PAID^a^ ≥ 40) among the study population (n = 255)

			Study population (n = 255)	Elevated diabetes-specific emotional distress	Low diabetes-specific emotional distress
**MDD** **^b^**			146 (57)		
		Anxiety^c^	69 (27)	46 (18)	23 (9)
		No anxiety	77 (30)	34 (13)	43 (17)
**No MDD**			109 (43)		
		Anxiety	26 (10)	17 (7)	9 (4)
		No anxiety	83 (33)	30 (12)	53 (21)
	Total			127 (50)	128 (50)

^a^ Problem Areas In Diabetes scale.

^b^ Major depressive disorder measured with the computerized version of the World Health Organization Composite International Diagnostic Interview (WHO CIDI-auto).

^c^ Anxiety disorder (WHO CIDI-auto).

Half of our study sample (n = 127, 49.8%) had elevated DM-distress. The patients with elevated DM-distress were younger, with a mean (SD) age of 47 (3) versus 53 (12), *P* < .001; were likelier to be female (85/127, 67% vs 70/128, 55%, *P* = .045); had higher baseline depression scores of mean (SD) CES-D 31 (7) vs 26 (6), *P* < .001; and were more likely to have an anxiety disorder (63/127, 50% vs 32/128, 25%, *P* < .001) than those without elevated DM-distress.

### Potential Effect Modifiers of the Treatment Effect

GEE analysis showed that having a diagnosis of MDD (*P* = .49) was not a significant effect modifier in the treatment effect on depressive symptoms (Table 3). In other words, we did not find significant differences in reduction of depressive symptoms for the intervention group versus control group, for patients with MDD compared with patients without MDD. This is after correcting for baseline differences in type of diabetes, use of antidepressant medication, depressive symptoms, and DM-distress, and the time between pre- and posttreatment.

**Table 3:** Intention-to-treat analyses (n = 125/130) of effectiveness of a Web-based diabetes-specific depression therapy on symptoms of depression as assessed by a CES-D^a^ score, testing effect modification by depression status, anxiety disorder, or high level of diabetes-specific emotional distress, in a cognitive behavioral therapy (CBT) intervention versus waiting-list (WL) control group^b^

	Pretreatment		Post treatment		1-month follow-up		*P* value
	CBT	WL	CBT	WL	CBT	WL	
MDD^c,d^	30 (7)	30 (7)	21 (11)	24 (9)	20 (12)	24 (10)	.49
No MDD	27 (7)	26 (7)	18 (9)	21 (8)	19 (10)	20 (8)	
Anxiety disorder^d^	32 (7)	31 (8)	23 (11)	25 (9)	22 (11)	25 (10)	.71
No anxiety disorder	27 (7)	26 (6)	19 (10)	21 (8)	19 (11)	21 (8)	
Elevated diabetes-specific emotional distress (PAID ≥40)^e^	31 (7)	31 (8)	22 (11)	24 (9)	21 (12)	24 (9)	.92
No elevated diabetes-specific emotional distress (PAID >40)^e^	26 (7)	26 (6)	18 (10)	22 (9)	18 (10)	21 (9)	

^a^ Center for Epidemiologic Studies Depression scale.

^b^ Data are given as mean (SD). Statistical tests relied on generalized estimating equation analyses. *P* values indicate level of significance of effect modification. All analyses are adjusted for baseline CES-D scores, baseline between-group differences on sociodemographic variables, and differences in time between pretreatment and posttreatment. Data are uncorrected.

^c^ Major depressive disorder.

^d^ Diagnosed using the computerized version of the World Health Organization Composite International Diagnostic Interview (WHO CIDI-auto).

^e^ Problem Areas In Diabetes scale.

Similarly, GEE analysis revealed that having an anxiety disorder diagnosis was not a significant effect modifier (*P* = .71) (Table 3). This is after correcting for baseline differences in depressive symptoms and DM-distress, and the time between pre- and posttreatment.

Also, having elevated DM-distress (*P* = .92), was not a significant effect modifier in the treatment effect on depressive symptoms (Table 3), after correcting for age, gender, baseline depression scores, baseline diagnosis anxiety disorder, and the time between pre- and posttreatment.

## Discussion

In this study, we aimed to answer the following questions: does the effectiveness of a Web-based diabetes-specific CBT depression intervention differ (1) for patients with or without MDD, (2) for patients with or without an anxiety disorder, and (3) for patients with or without elevated DM-distress? Secondary analyses from an RCT comparing Web-based diabetes-specific depression treatment versus a waiting-list control in patients with type 1 and type 2 diabetes with comorbid depression show that a diagnosis of MDD or anxiety disorder, or reporting high DM-distress does not significantly modify the effect of Web-based diabetes-specific CBT depression treatment. These findings thus suggest that there is no reason to exclude patients with more severe depression or anxiety from participating in what is often considered a first line of treatment for patients with mild depression, following a stepped-care approach [28]. When referring patients to this Web-based, diabetes-specific depression treatment, screening for depressive symptoms using a questionnaire seems advisable, omitting the need to strictly diagnose MDD. It is at this stage unclear for whom the intervention is contraindicated. In our study, patients were excluded in case of psychotic features, suicidal ideation or a history of suicide attempts, or previous admission to a psychiatric hospital for depression treatment. It seems advisable, at minimum, to check for suicidal ideation and psychotic features as exclusion criteria.

Interestingly, roughly half of the participating diabetes patients with comorbid depression reported high disease-specific distress, which was found not to be an effect modifier. This suggests that DbG.nl is suitable for patients with and without high diabetes-related distress. In a future study, comparing the diabetes-specific intervention with a generic Web-based depression intervention on effectiveness and attractiveness from the patient’s perspective would be of great importance.

In interpreting the results of our study, we should acknowledge several strengths and limitations of the study. The most important strengths are the design of the study, being an RCT, and the innovative character of the study. This is the first study that tested the effectiveness of Web-based, diabetes-specific depression treatment in different subgroups of patients. Moreover, we administered a diagnostic interview and used validated instruments for measuring depressive symptoms and DM-distress.

An important limitation that needs mentioning is that examining the effect modification was not the primary aim of our RCT. Therefore, this study was not powered to detect significant differences in effect between patients with and without MDD, anxiety disorder, and elevated DM-distress. Yet, due to the relatively large sample size of the study, we had substantial subgroups of patients to compare (n = 146 patients with MDD, n = 109 patients with anxiety disorder, and n = 127 patients with elevated DM-distress). We were unable to test whether specifically MDD was more difficult to treat when patients had comorbid anxiety, but instead examined the effect modification in the full sample of subclinically depressed and MDD patients. Since in our study sample a substantial group had both MDD and anxiety disorder, this stresses the importance of testing the effect modification of anxiety disorder in a sample of diabetes patients with MDD.

Regarding external validity of our results, we should take into account that our sample consisted largely of white, relatively well-controlled diabetes patients, including only a few less-educated people (about 8/255, 3%) and patients older than 65 years (24/255, 9%). Considering the increased prevalence of diabetes in ethnic populations, older people, and those with lower social economic status, further research is warranted to test the effectiveness of our program in more diverse populations. We also observed that only a few of the participants with MDD had recurrent depression, even though MDD has been shown to be more recurrent in diabetes patients [29]. Perhaps the underrepresentation of patients with recurrent depressive episodes in our study can be explained by these patients already having tried several forms of depression treatment and, and therefore being less willing to try a new form of therapy (Web-based therapy). Future studies should make an effort to attract diabetes patients with recurrent depression in order to test the effectiveness of Web-based therapy.

### Conclusions

Findings from this study provide the first evidence to suggest that Web-based diabetes-specific depression treatment is effective in patients with mild to more severe depression, with or without comorbid anxiety disorder. Information on differential effects of Web-based therapy is vital to make evidence-based recommendations regarding indication, referral, and reimbursement of the intervention.

The Web-based, diabetes-specific depression treatment seems to have high usability because it can serve as an intervention for both subclinical and clinical depression in diabetes patients. Given its Web-based administration, this diabetes-specific depression treatment has the potential to reach large patient populations and to be cost effective.

## Abbreviations

CBT: cognitive behavioral therapy
CES-D: Center for Epidemiologic Studies Depression scale
DbG.nl: Diabetergestemd.nl
DM-distress: diabetes-specific emotional distress
GEE: generalized estimating equation
MDD: major depressive disorder
PAID: Problem Areas in Diabetes scale
RCT: randomized controlled trial
WHO CIDI-auto: computerized version of the World Health Organization Composite International Diagnostic Interview
